# Clinical exome sequencing in daily practice: 1,000 patients and beyond

**DOI:** 10.1186/gm521

**Published:** 2014-01-24

**Authors:** Wendy A van Zelst-Stams, Hans Scheffer, Joris A Veltman

**Affiliations:** 1 Department of Human Genetics, Nijmegen Centre for Molecular Life Sciences, Institute for Genetic and Metabolic Disease, Radboud University Medical Centre, 6500 HC, Nijmegen, The Netherlands

## Diagnostic exome sequencing

Using next-generation sequencing technology to sequence the coding regions of all human genes, known as exome sequencing, has rapidly become one of the most successful methods for genetic disease research since its introduction in 2009 [1]. The possibility to investigate all genes for mutations in a single experiment holds great promise in the diagnostic arena, in particular for genetically heterogeneous disorders and clinically undiagnosed diseases. Laboratories, including our own, have now started to offer exome sequencing as a diagnostic test. In this article, we share our experience with diagnostic exome sequencing and discuss the impact of these novel diagnostic methods on medical practice, now and in the future.

## What have we learned so far?

We set up exome sequencing in our diagnostic laboratory, which is quality accredited in compliance with International Organization for Standardization (ISO) standard 15189 (similar to Clinical Laboratory Improvement Amendments accreditation), and patient inclusion and counseling were coordinated by our clinical genetics unit, both located within the Department of Human Genetics of the Radboud University Medical Center. We immediately noted a high demand from referring clinicians for exome sequencing because of the number of unsolved genetic diseases. We initially had to restrict patient inclusion because of limited sequencing capacity and high costs; we also wanted to build up experience in clinical interpretation. Inclusion was also restricted to referral by in-house clinical geneticists so that adequate genetic counseling could be provided to patients before and after this novel test was performed, as well as allowing us to learn from unexpected findings and easily adapt procedures.

We encountered no major hurdles while setting up the highly automated exome sequencing in the laboratory, making use of our unit’s extensive experience with this approach. More than 1,000 diagnostic exomes have been sequenced so far in our department, and we are now scaling up this approach to many more genetic diseases in collaboration with our partners at BGI Europe. Much of the workload in exome sequencing has shifted from the wet laboratory to the dry laboratory, specifically the data analysis and subsequent interpretation and reporting. This requires a dedicated team of computational scientists working together with clinical laboratory geneticists and clinical geneticists. Interpretation of the pathogenicity of variants is greatly facilitated by the rapidly expanding reference databases of genetic variation, such as the National Center for Biotechnology Information Single Nucleotide Polymorphism Database and the National Heart, Lung, and Blood Institute exome variant server. However, such interpretation requires expert knowledge and careful use of these normal variation frequencies in combination with pathogenicity prediction tools and other useful annotations.

One of the main issues we found in counseling patients prior to exome sequencing involved the potential identification of unsolicited, incidental or secondary findings - genetic variants that are medically relevant but not for the disease for which the patients visited the clinic [2]. It was unclear at the start of this endeavor how frequently such a variant would be found during the diagnostic process, and what kind of variants would be observed. Our previous experience with non-targeted diagnostic approaches was limited to genome-wide array-based diagnostics and classical cytogenetics. Many clinicians feared that the number of unsolicited findings would be very high and do more harm than good to the patients, and complicate diagnostic procedures. We therefore decided from the start to establish an independent expert panel that could determine the clinical relevance of such unsolicited findings, to counsel all patients and families involved about the possibility of unsolicited findings and consent them to being informed if the expert panel determined the findings clinically relevant, and to restrict diagnostic interpretation as much as possible to mutations in known disease-causing genes.

A list of known disease-causing genes was made for each condition tested by exome sequencing. Variants outside these genes were automatically filtered out during the data analysis. This minimized the risk of unsolicited findings and at the same time selected for exome variation that was most likely to explain a particular disease, an approach that we have used successfully for disorders like blindness, deafness, movement disorders, hereditary cancer and mitochondrial disorders. For most of the disorders studied, with the exception of microsatellite stable colorectal cancer, the diagnostic yield of exome sequencing was clearly superior to that of traditional sequential gene testing, reaching up to 52% for patients with blindness [3]. Importantly, we learned that the phenotypic definition of a disease and associated gene list should not be too restricted, as disease genes may have a much broader phenotypic spectrum than previously reported.

For intellectual disability, which mostly occurs sporadically and for which the majority of disease genes are presently unknown, we took a different strategy. We focused on *de novo* mutations as well as recessive mutations occurring throughout the exome, using a patient-parent trio sequencing approach [4]. This has resulted in a diagnostic yield of at least 16% in this well-studied patient group, with an additional 22% of patients harboring mutations predicted to be pathogenic in strong candidate genes [5]. This approach obviously does not restrict the analysis to genes already implicated in a particular clinical entity. It therefore also leads to a higher chance of identifying unsolicited variants such as *de novo* mutations in cancer predisposition genes. In our first series of 100 patients, we identified a *de novo* mutation in *RB1*, associated with retinoblastoma, in a patient with severe intellectual disability. The expert panel considered the risk of retinoblastoma to be negligible for this patient, given that he had reached the age of 8 years with no tumor occurrence, but decided that it was important to inform the parents of the small chance that a sudden, painful swelling of the limbs could be caused by an osteosarcoma and that they should consult an oncologist at the first sign of such swelling.

International discussion on the reporting of unsolicited findings has, meanwhile, intensified. The American College of Medical Genetics, among others, recommends that a limited number of medically relevant variants should be specifically sought and reported in each patient undergoing exome or genome sequencing [6]. We are actively involved in these discussions and agree with recent recommendations by the European Society of Human Genetics that unsolicited genetic variants indicative of treatable or preventable health problems should be reported [7].

One of the major remaining issues is now to reduce the turnaround time from months to weeks, so referring clinicians can rely on exome sequencing as a rapid and robust diagnostic approach. This is now a realistic possibility because of the increased speed, throughput and quality of the latest next-generation sequencing platforms, in combination with faster data-analysis pipelines. Still, it is important to note here that current exome sequencing is far from perfect and should be further improved [8]. Many exons are not enriched and/or sequenced well, insertion-deletions as well as structural variation are only partially detected and homologous sequences cause problems in the correct mapping of sequencing reads. It is therefore clear that, at present, it is not the best test available to exclude pathogenic mutations in a specific gene or set of genes. If this is required, we currently recommend deep sequencing of relevant gene panels. In addition, exome sequencing is not the preferred method for the detection of other mutational mechanisms such as trinucleotide expansions or imprinting defects. Finally, Sanger sequencing validation of all clinically relevant variants is still highly recommended at this time.

## Impact of exome and genome sequencing on medical practice

Although technological improvements are essential for the widespread clinical implementation of exome sequencing, we have no doubt that these will be developed in the coming years. It will not be long before exome and, not much later, genome sequencing become standard tools in each genetic laboratory. We anticipate that it will not be too much longer before exome or genome data are available to a clinician within hours to minutes, and therefore can be used during the first clinical evaluation of a patient. This rapid availability of genomic information is crucial for genetics to play a more central role in modern medicine, as time is of primary importance in clinical decision-making. An example of this was recently given for applications of rapid whole-genome sequencing to diagnose rare genetic diseases in neonatal intensive care units [9].

Affordable and rapid availability of exome or genome information will also have a major impact on the role of both the clinical geneticist and the laboratory specialist. Traditionally, an important role of the clinical geneticist was to request the best genetic test for confirmation of a clinical diagnosis at the molecular level. This request was based on expert knowledge in clinical phenotyping of mostly rare genetic diseases after other medical specialists had excluded more common causes. The laboratory specialist would perform these tests and interpret the data. However, in the near future, the clinical geneticist will be involved in the first clinical examination of a patient in the case of many diseases as an expert who can rapidly link a patient’s phenotype with his or her genotype, both of which are in front of the clinician. The laboratory specialist will support the clinical geneticist in this process, and perform additional tests to confirm pathogenicity of individual genomic variants or study more complex modes of inheritance. Increasingly, genetics will be used to determine the right therapy and for monitoring treatment efficacy. This futuristic scenario is closer than many of us may think, and will require a change in our laboratory and clinical training programs and a further integration of genetics in the clinic. A first important step towards these goals is the implementation of exome sequencing as a first-tier test for genetically heterogeneous disorders.

## Competing interests

The authors declare that they have no competing interests.
